# Methodological Underestimation of Oceanic Nitrogen Fixation Rates

**DOI:** 10.1371/journal.pone.0012583

**Published:** 2010-09-03

**Authors:** Wiebke Mohr, Tobias Großkopf, Douglas W. R. Wallace, Julie LaRoche

**Affiliations:** Marine Biogeochemie, Leibniz-Institut für Meereswissenschaften (IFM-GEOMAR), Kiel, Germany; Mt. Alison University, Canada

## Abstract

The two commonly applied methods to assess dinitrogen (N_2_) fixation rates are the ^15^N_2_-tracer addition and the acetylene reduction assay (ARA). Discrepancies between the two methods as well as inconsistencies between N_2_ fixation rates and biomass/growth rates in culture experiments have been attributed to variable excretion of recently fixed N_2_. Here we demonstrate that the ^15^N_2_-tracer addition method underestimates N_2_ fixation rates significantly when the ^15^N_2_ tracer is introduced as a gas bubble. The injected ^15^N_2_ gas bubble does not attain equilibrium with the surrounding water leading to a ^15^N_2_ concentration lower than assumed by the method used to calculate ^15^N_2_-fixation rates. The resulting magnitude of underestimation varies with the incubation time, to a lesser extent on the amount of injected gas and is sensitive to the timing of the bubble injection relative to diel N_2_ fixation patterns. Here, we propose and test a modified ^15^N_2_ tracer method based on the addition of ^15^N_2_-enriched seawater that provides an instantaneous, constant enrichment and allows more accurate calculation of N_2_ fixation rates for both field and laboratory studies. We hypothesise that application of N_2_ fixation measurements using this modified method will significantly reduce the apparent imbalances in the oceanic fixed-nitrogen budget.

## Introduction

Biological dinitrogen (N_2_) fixation is the major source of fixed nitrogen (N) in the oceanic N budget [1]. Current estimates of global oceanic N_2_ fixation are ∼100–200 Tg N a^−1^ [2]. N_2_ fixation rates can be assessed through geochemical estimates, modelling of diazotroph abundances and growth rates [3] and direct measurements of N_2_ fixation. Geochemical estimates rely on the measurement of, *e.g.*, nutrient stoichiometry and estimates or models of ocean circulation [4], [5] or the distribution of stable isotope abundances (*e.g.*, [6]). Direct measurements of N_2_ fixation are obtained either using the ^15^N_2_-tracer addition method [7], [8] or the acetylene reduction assay (ARA) [8], [9]. However, direct measurements of N_2_ fixation rates account for ≤50% of the geochemically-derived estimates [10]. Furthermore, the sink terms in the oceanic fixed N budget significantly exceed the current estimates of N_2_ fixation and other source terms for fixed N [11], [12]. This gap between sources and sinks of fixed N implies an oceanic nitrogen imbalance, which may reflect a non-steady-state of the oceanic fixed-N inventory, or result from over-estimation of loss processes and/or under-estimation of fixed nitrogen inputs [10], [13]. However, isotopic signatures in sediments suggest that the fixed N budget is in a steady-state [14].

The comparison of N_2_ fixation rates measured simultaneously using the ^15^N_2_-tracer addition and the ARA shows that the ^15^N_2_-tracer addition generally yields lower rates (for a summary see [15]). In addition, mass balance analyses of ^15^N_2_-based N_2_ fixation rates measured in experiments with cultured diazotrophs, indicate that the ^15^N_2_-tracer addition method yields rates that are too low for sustaining the observed growth rates and biomass [16], [17]. The discrepancies between the two methods and the lack of mass balance in culture experiments have often been attributed to the excretion of recently fixed nitrogen as ammonium (NH_4_^+^) or dissolved organic nitrogen (DON). The discrepancies have led to the operational definition of gross and net N_2_ fixation [16], [18] as measured by the ARA and the ^15^N_2_-tracer addition approaches, respectively. However, the measured release of NH_4_^+^ or DON is rarely sufficient to balance the observed growth in culture, and even invoking recycling of the dissolved fixed N rarely accounts for the observed discrepancies between N_2_ fixation rate and growth rate/biomass [16].

The apparent oceanic N imbalance, differences between geochemical estimates and measured rates of N_2_ fixation, and the difficulties in reconciling discrepancies between ARA and ^15^N_2_-based estimates of N_2_ fixation in the field and in culture experiments, led us to re-assess the ^15^N_2_-tracer addition method. This method is based on the direct injection of a ^15^N_2_ gas bubble into a seawater sample [7] sufficient to yield a final enrichment of 2–5 atom percent (atom%) and incubation for 2–36 hours [19]. N_2_ fixation rates are then retrieved from the incorporation of ^15^N_2_ into the particulate organic nitrogen (PON). The method assumes implicitly that the injected gas fully and rapidly equilibrates with the surrounding water, and this assumption is the basis for calculation of the initial ^15^N enrichment of the dissolved N_2_ pool. Knowledge of this enrichment is pivotal to the calculation of N_2_ fixation rates with this method as seen in equation 1 (equations combined from [8]):
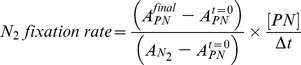
where A = atom% ^15^N in the particulate organic nitrogen (*PN*) at the end (*final*) or beginning (*t = 0*) of the incubation or in the dissolved N_2_ pool (*N_2_*).

In applications of the method, all parameters of the equation are measured except for the atom% ^15^N in the dissolved N_2_ pool (*A_N2_*). Equation 1 shows that calculation of N_2_ fixation rates depends strongly on this value which is calculated from the predicted equilibrium dissolved N_2_ concentration [20], [21], its natural ^15^N abundance, and the amount of ^15^N_2_ tracer added with the bubble. The calculation assumes that there is complete isotopic equilibration between the injected bubble of ^15^N_2_ and the surrounding water at the start of the incubation.

Here we report results of experiments that were designed to assess the rate of equilibration of an introduced ^15^N_2_ gas bubble with the surrounding water. Based on results of these experiments, we developed a modified approach involving addition of ^15^N_2_-enriched seawater which assured a well-defined and constant ^15^N enrichment of the dissolved N_2_ gas at the beginning of the incubations. We propose the application of the modified approach for future assessments of N_2_ fixation rates in natural microbial communities and in laboratory cultures.

## Results

### Time-resolved equilibration of a bubble of ^15^N_2_ in seawater

**Figure 1:**
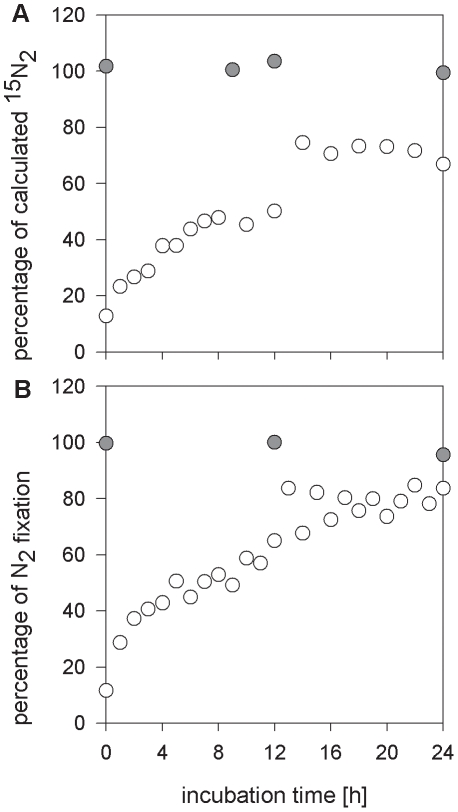
Time-dependence of the equilibration of a ^15^N_2_ gas bubble with seawater. Results are presented as a function of the time after bubble injection (white symbols). (A) Measured dissolved ^15^N_2_ concentrations as percentage of calculated concentration assuming rapid and complete isotopic equilibrium. (B) N_2_ fixation rates by *C. watsonii* as percentage of the maximum rate measured during the experiments. For comparison, the addition of ^15^N_2_-enriched water to samples yielded a constant ^15^N_2_ enrichment over 24 h (A, grey symbols) or constant N_2_ fixation rates (B, grey symbols).

A first set of experiments (isotopic equilibration experiments) was carried out to assess the time required to attain isotopic equilibrium in the dissolved pool of N_2_ gas after injection of a known amount of ^15^N_2_ gas as a bubble into sterile filtered seawater. A gas bubble of pure ^15^N_2_ was injected directly into incubation bottles which were manually inverted fifty-times (∼3 min agitation) and left standing for up to 24 h. Concentration of dissolved ^15^N_2_ was followed over the 24 h period to assess the degree of equilibration of the ^15^N_2_ gas bubble with the surrounding water as a function of time. Dissolved ^15^N_2_ concentrations in the seawater increased steadily with the incubation time (Fig. 1A). After eight hours, dissolved ^15^N_2_ concentrations reached about 50% of the concentration calculated assuming complete isotopic equilibration of the injected bubble with the ambient dissolved N_2_ gas in the seawater sample. At the end of the 24 h incubation, the dissolved ^15^N_2_ concentration had increased to about 75% of the calculated concentration.

### N_2_ fixation rate underestimation due to incomplete ^15^N_2_ gas bubble equilibration

Similar results were obtained in the incubation experiments with pure culture of *Crocosphaera watsonii* (culture experiments), which confirmed the incomplete and time-dependent equilibration of the injected bubble of ^15^N_2_ gas with the surrounding water (Fig. 1B). These experiments also demonstrated the associated underestimation of N_2_ fixation rates. Culture experiments were conducted after ^15^N_2_ addition as a gas bubble and also after ^15^N_2_ addition in the form of ^15^N_2_-enriched seawater (our modified method, see Methods section). The incubation of *C. watsonii* after injection of a bubble of ^15^N_2_ gas and without prior incubation of this bubble in algal-free media, gave a N_2_ fixation rate which was only 40% of the maximum rate measured in the incubations to which ^15^N_2_-enriched seawater had been added. In other words, for the 12-h incubation period under the described experimental conditions, the N_2_ fixation rate was underestimated by 60% when the ^15^N_2_ was introduced as a gas bubble. In contrast, in both the isotopic equilibration and the culture experiments, the concentration of dissolved ^15^N_2_ remained stable at the predicted value throughout the 24 h in incubations to which ^15^N_2_-enriched water was added.

### Factors influencing ^15^N_2_ gas dissolution in N_2_-saturated seawater

**Figure 2:**
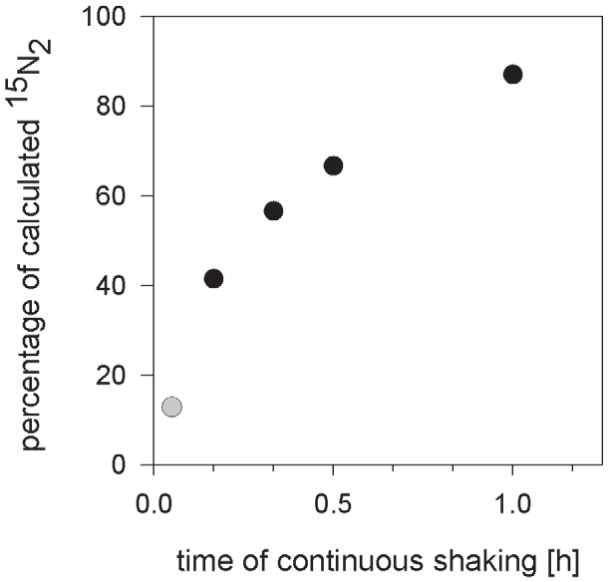
Agitation-dependent increase in dissolved ^15^N_2_ using bubble incubations. Values are presented as a percentage of the calculated concentration. The manually-shaken (3 min) sample was added to the plot for comparison (grey symbol).

Continuous, vigorous shaking (50 rpm) greatly increased the concentration of ^15^N_2_ in the media (Fig. 2) reaching ∼67% of the calculated concentration after 30 minutes whereas the initial, manual agitation, *i.e.* inverting bottles 50 times (∼3 min), resulted in only ∼13% of the calculated concentration. Information on agitation is generally not provided in the published literature, but this is clearly a variable factor in incubations, especially if performed at sea. Continuous, vigorous shaking, as tested here (50 rpm; Fig. 2), is difficult to achieve in field experiments and may, in addition, be detrimental to some diazotrophs (*e.g. Trichodesmium* colonies).

**Figure 3:**
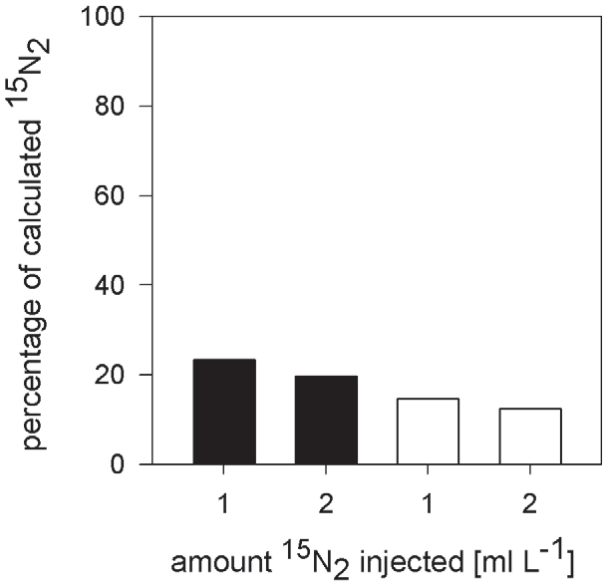
Dissolved ^15^N_2_ concentration as a function of bottle size and amount of injected ^15^N_2_ gas. Values are presented as a percentage of the calculated concentration. Bottles were incubated for 1 hour. Black bars, 0.13 L bottle and white bars, 1.15 L bottle.

**Figure 4:**
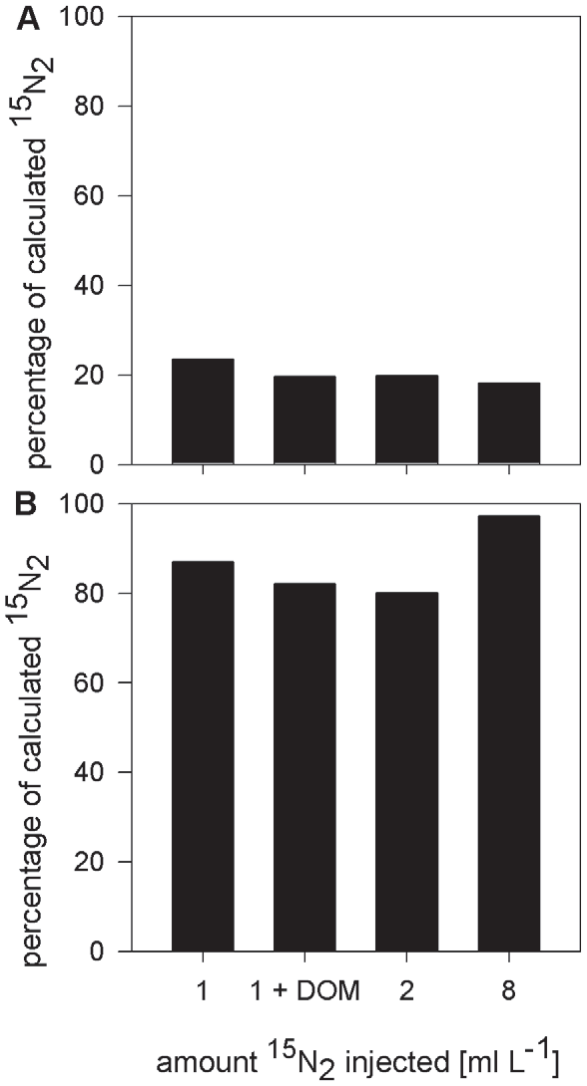
Dissolved ^15^N_2_ concentration as a function of the amount of injected gas and agitation. Values are presented as a percentage of the calculated concentration (A) after 1 hour incubation in manually (3 min shaking and 1 h subsequent incubation), and (B) in continuously (1 h) shaken samples.

Increasing the size of the incubation bottles, increasing the amount of gas injected per liter of seawater and the addition of dissolved organic matter (DOM) led in all cases except one to slower equilibration of the ^15^N_2_ gas bubble with the surrounding water (Fig. 3 and 4A), even when bottles were shaken continuously for one hour (Fig. 4B). Only with the injection of 8 ml of ^15^N_2_ gas per liter of water and one hour of continuous, vigorous shaking, was near-complete equilibration achieved (97% of calculated concentration).

## Discussion

Both the isotopic equilibration and the culture experiments demonstrated clearly that the equilibration of ^15^N_2_ gas injected as a bubble into N_2_-saturated seawater is time-dependent and incomplete, even after 24 hours. The lack of complete equilibration causes the resulting calculated N_2_ fixation rates to be variably and significantly underestimated (see Equation 1). The equilibration, *i.e.* the isotopic exchange between the ^15^N_2_ gas in the bubble and the surrounding water is controlled primarily by diffusive processes. The major variables that influence the rate of isotopic exchange include the surface area to volume ratio of the bubble, the characteristics of the organic coating on the bubble surface [22], temperature and the rate of renewal of the water-bubble interface [23]. The renewal of the water-bubble interface appears to have the greatest effect on the isotopic exchange, as continuous vigorous shaking of the incubation bottles generated the highest enrichment of ^15^N_2_ in the water phase. However, the calculated (equilibrium) enrichment in ^15^N_2_ was not attained fully even after one-hour of continuous shaking at 50 rpm on a rotary shaker. Incubations carried out on board a research vessel will provide some agitation of the bubble but this will not approach the high and constant agitation tested in our experiments. The implication is that variable sea-state conditions encountered during sea-going incubations, and the details of individual experiments, will lead to variable ^15^N_2_ enrichments and hence variable underestimation of N_2_ fixation rates. Further, N_2_ fixation studies in the oligotrophic regions of the ocean usually require the use of large incubation volumes (*e.g.*, 2–4 L), so that continuous shaking for one hour or more is not practical, and in addition would likely be detrimental to the natural microbial communities.

The experiments with variable bottle sizes and DOM additions (Fig. 3 and 4) demonstrated that there are factors in addition to the bubble incubation time that affect the equilibration. On the other hand, the addition of ^15^N_2_-enriched seawater to the incubations led to a stable enrichment over the 24 h incubation time which was instantaneous and independent of the agitation of the bottles.

This study was motivated partly by the mismatches between the ARA and ^15^N_2_-based measurements of N_2_ fixation as well as imbalances between ^15^N_2_-fixation rates and biomass-specific rates (∼growth rate) or C∶N fixation ratios (Table 1). Such mismatches have been observed in environmental studies and in culture studies, mainly with *Trichodesmium*. Although it has been shown that *Trichodesmium* can excrete recently fixed N_2_ as NH_4_^+^ or DON [16], [24], the excretion of ^15^NH_4_^+^ or DO^15^N rarely accounts for the observed discrepancies [16], [17]. The operational definition of gross and net N_2_ fixation as obtained through ARA and ^15^N_2_ incubations, respectively, has been mainly based on the mismatch between the rates measured by the two methods. Our results demonstrate that N_2_ fixation rates, as measured with the ^15^N_2_ method [7] are underestimated. Therefore, the magnitude of the exudation of recently fixed nitrogen and the conditions promoting this process should be re-evaluated, taking into account the results presented here.

**Table 1:** Discrepancies observed between ^15^N_2_ fixation, ARA and carbon fixation or biomass-specific rates^a^.

Organism/area	C_2_H_2_∶^15^N_2_	C∶N fixation ratio	biomass-specific rate [d^−1^]	Reference
*Trichodesmium*/Pacific, Atlantic, north of Australia		808^b^		[30]
cyanobacterial bloom/Baltic	3–20			[18]
*Trichodesmium* IMS 101	3–22	75–133		[17]
*Trichodesmium* IMS 101	1.5–6.9		0.002–0.011^c^	[16]
*Trichodesmium*/Gulf of Mexico		10–107^b^		[31]
*Trichodesmium*/Bermuda Atlantic Time Series station (BATS)		13–437	0.006–0.03^d^	[32]

a C∶N fixation ratio is based on ^15^N_2_-fixation measurements.

b Ratio calculated from DI^13^C and ^15^N_2_ fixation rates.

c Calculated from ^15^N_2_ fixation rate divided by PON.

d Calculated from doubling time with biomass-specific rate = ln (2)/doubling time.

**Figure 5:**
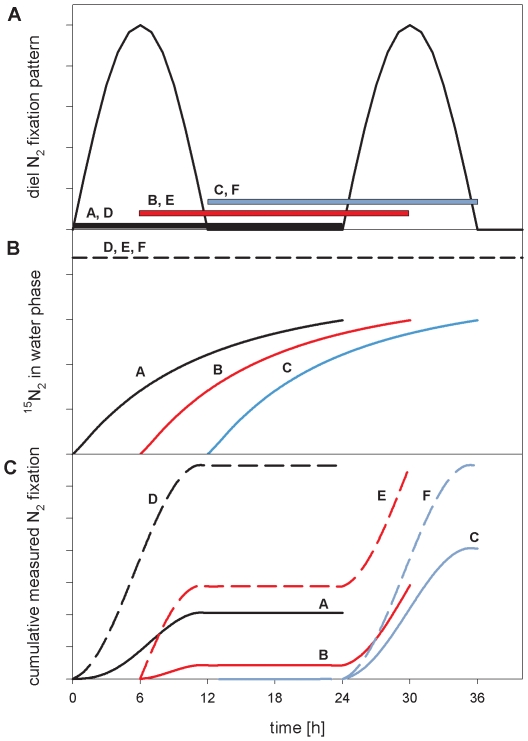
Influence of diel N_2_ fixation patterns on the magnitude of N_2_ fixation rates. Schematic diagram illustrating the influence of diel N_2_ fixation patterns on N_2_ fixation rates when determined with the direct injection of a ^15^N_2_ gas bubble. A hypothetical diel N_2_ fixation pattern is shown (panel A) with a duration of the N_2_-fixing period of 12 h. Three possible time periods for 24 h incubations are indicated by the solid bars (A–F). The corresponding ^15^N enrichment in the dissolved N_2_ pool (panel B) is shown for the three incubation periods using the direct injection of a ^15^N_2_ gas bubble (solid lines; A, B and C) and the addition of ^15^N_2_-enriched seawater (dashed line; D, E and F). The resulting cumulative N_2_ fixation in each of the incubations (panel C) demonstrates that the timing of the incubation relative to diel N_2_ fixation patterns introduces a variable underestimation in the total N_2_ fixation rate measured during the incubation after a ^15^N_2_ gas bubble is injected (solid lines; A, B and C) as compared to the N_2_ fixation measured with the addition of ^15^N_2_-enriched seawater (dashed lines; D, E and F). The diagram is based on the observations made in the experiments described in this study.

We reviewed published studies that have used the direct injection of a ^15^N_2_ gas bubble to assess N_2_ fixation rates in order to evaluate the magnitude of under-estimation. However, first attempts to assess the degree of underestimation of field and culture N_2_ fixation rates were obscured by a wide range of experimental conditions among the studies. Bottle sizes ranged from 14 ml to 10 L, the amount of ^15^N_2_ injected varied from 0.2 to 40.8 ml ^15^N_2_ per L seawater and incubation times ranged from 0.25 to 48 hours, with the majority of the field studies using 2–4 L bottles and 24 h incubations. In addition, information on agitation was, in general, not available. There were no obvious trends of reported N_2_ fixation rates with either bottle size, incubation time or the amount of injected ^15^N_2_ gas probably because of the large variability of geographic locations and environmental conditions prevailing in the individual studies, which would have a dominant effect on the local diazotrophic communities and their N_2_ fixation rates. An evaluation of the degree of possible underestimation of ^15^N_2_ fixation rates in environmental studies is further confounded by diel periodicity of N_2_ fixation [25]–[27]. The lack of knowledge on the exact timing and magnitude of the individual N_2_ fixation activity of the different diazotrophs relative to the timing of ^15^N_2_ gas injection hinders back-calculation of published N_2_ fixation data. This can be illustrated, for example, with a hypothetical diazotroph community that is dominated by unicellular cyanobacteria which fix nitrogen during the night period only (Fig. 5A). In this microbial community, measurements of N_2_ fixation using the direct injection of a ^15^N_2_ gas bubble during a 24 hour incubation will lead to a variable underestimation of the true N_2_ fixation rate, depending on the timing of the incubation start relative to the peak in the nitrogenase activity (Fig. 5C, solid lines). The underestimation will be more pronounced if the start of the incubation is coincident with the onset of the active N_2_ fixation period. In contrast, incubations with enriched ^15^N_2_ seawater, will not lead to an underestimate, regardless of the incubation start relative to the diel cycle (Fig. 5C, dashed lines).

The discrepancies and mismatches/imbalances observed in field and laboratory studies could, in part, be explained by the variable underestimation of the true N_2_ fixation rate due to the methodological uncertainty reported here. We propose the addition of ^15^N_2_-enriched seawater to incubations to assess N_2_ fixation rates in laboratory and field studies. We suggest that measurements using this approach are likely to increase measurements and estimates of N_2_ fixation at species, regional and global level and lead to a reduction in the apparent oceanic nitrogen imbalance.

## Materials and Methods

### Culture and growth conditions

The diazotrophic cyanobacterium *Crocosphaera watsonii* WH8501 was grown in batch cultures in N-free YBCII media [28] at 28°C in a temperature-controlled growth chamber. *C. watsonii* was subjected to 12∶12 h dark∶light cycles.

### Direct injection of a ^15^N_2_ gas bubble in water

We first examined the rate of equilibration between an injected bubble of ^15^N_2_ gas and seawater. Two series of incubations were started by injecting 140 µl of ^15^N_2_ into 133 ml of an artificial seawater media (YBCII) contained in headspace-free, septum-capped glass bottles. In the first series (isotopic equilibration experiments), all bottles were inverted fifty times (∼3 min) after injection of the ^15^N_2_ gas bubble and left at room temperature in the laboratory. One bottle was sampled immediately after the agitation in order to determine how much ^15^N_2_ gas had dissolved initially. The other bottles were opened and sampled after standing for periods from 1 to 24 h. Upon opening of the bottles, samples to measure the dissolved ^15^N_2_ were taken and stored in gas-tight glass vials (Exetainer®) until analysis.

In the second series (culture experiments), the YBCII media was pre-heated to 28°C in a temperature-controlled chamber before being used to fill septum-capped glass bottles. As with the first series, samples were agitated and left standing for varying periods of time after the injection of a ^15^N_2_ gas bubble. Instead of taking subsamples for ^15^N_2_ analysis, 13 ml of media were replaced by *C. watsonii* WH8501 culture upon opening of the bottles. This series of experiments was timed so that the introduction of culture into the media took place at the start of a dark phase of the 12∶12h dark∶light-adapted *C. watsonii* culture. The samples with the culture were then incubated for 12 h at culture growth conditions (28°C, dark phase, *i.e.* N_2_-fixing) and filtered onto pre-combusted GF/F (Whatman; 450°C for 4 h) filters at the end of the incubation. Filters were dried immediately after (50°C, 6 h) and stored at room temperature until analysis. To obtain a measure of underestimation using the direct injection of a ^15^N_2_ gas bubble, one bottle containing 13 ml of *C. watsonii* culture was incubated for 12 h after the injection of 140 µl ^15^N_2_ gas at the start of the dark phase and without release of the bubble, essentially resembling a laboratory or field incubation.

### Direct addition of ^15^N_2_ tracer-enriched seawater

An alternative, modified ^15^N_2_ tracer addition method was developed, which involved addition of an aliquot of ^15^N_2_-enriched water to incubations. This alternative method was based on earlier approaches used to study oxygen cycling using ^18^O_2_ [29] and the release of DON using ^15^N_2_ [24]. The preparation of the ^15^N_2_-enriched water was started by degassing 0.2 µm-filtered artificial seawater (YBCII media). Degassing was carried out by applying vacuum (≤200 mbar absolute pressure) to continuously stirred (stir bar) media for about 30 min. The degassed water was transferred rapidly but gently into septum-capped glass bottles until overflow, and 1 ml of ^15^N_2_ gas (98 at%; Campro Scientific) was injected per 100 ml of media. The bottles were shaken vigorously until the bubble disappeared. Aliquots of this ^15^N_2_-enriched water were then added to the incubation bottles, with the enriched water constituting no more than 10% of the total sample volume. This alternative enrichment method was applied to the two series of experiments described above.

### Assessment of additional factors contributing to variation in ^15^N_2_ enrichment

We assessed possible effects of varying bottle size, amounts of injected gas and different amounts of agitation on their contribution to the equilibration between a bubble of ^15^N_2_ gas and the surrounding seawater. For the bottle size comparison, incubations were performed in 0.13 L bottles and in 1.15 L bottles. The amount of injected gas varied between 1 ml ^15^N_2_ per 1 L seawater up to 8 ml ^15^N_2_ per 1 L seawater. The incubations were agitated either by inverting fifty times manually (∼3 min) or by continuous agitation on a rotating bench-top shaker (Biometra WT 17) at 50 rpm (rotations per minute). We also added marine broth (Difco 2216; 0.2 µm filter-sterilized; 230 mg DOM L^−1^ media) to some bottles to examine the effect of dissolved organic matter (DOM).

### ^15^N_2_ analysis in the artificial seawater and ^15^N analysis in the particulate organic nitrogen (PON)

Subsamples taken during the equilibration experiments were analysed for ^15^N_2_ concentration with a membrane-inlet mass spectrometer (MIMS; GAM200, IPI) within one week of subsampling. Dried GF/F filters were pelletized in tin cups, and PON as well as isotope ratios were measured by means of flash combustion in an elemental analyser (Carlo Erba EA 1108) coupled to a mass spectrometer (Thermo Finnigan Delta S).

### Calculations

The expected concentration of ^15^N_2_ following bubble injections was calculated assuming rapid and complete isotopic equilibration between bubble and surrounding seawater and considering atmospheric equilibrium concentrations of dissolved N_2_ [21]. When ^15^N_2_-enriched aliquots were added, the amount of ^15^N_2_ originally dissolved in the degassed seawater and the volume of the aliquot added were taken into account. The calculations of N_2_ fixation rates in the culture incubations were made according to Equation 1 and are presented as a percentage of the highest rate measured. For the comparison between methods, the measured ^15^N_2_ concentrations are presented as a percentage of the expected concentration calculated as follows
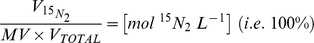
(2)for the direct injection of a ^15^N_2_ gas bubble where 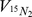 is the volume of the ^15^N_2_ gas bubble, *MV* is the molar volume and *V_TOTAL_* is the total (water) volume of the incubation. The expected concentration was corrected for the amount of ^15^N_2_ gas which remains in the bubble at isotopic equilibrium with the surrounding water. For the addition of ^15^N_2_-enriched water the expected concentration is
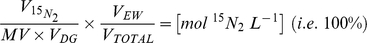
(3)where *V_DG_* is the volume of degassed water, *V_EW_* is the volume of ^15^N_2_-enriched water added to the incubation and *V_TOTAL_* is the total (water) volume of the incubation.
